# The unstable knee in congenital limb deficiency

**DOI:** 10.1007/s11832-016-0784-y

**Published:** 2016-11-08

**Authors:** Gabriel T. Mindler, Christof Radler, Rudolf Ganger

**Affiliations:** Department of Pediatric Orthopaedics, Orthopaedic Hospital Speising, Speisinger Strasse 109, 1130 Vienna, Austria

**Keywords:** Congenital longitudinal deficiency, Fibular hemimelia, Knee instability, Bone lengthening

## Abstract

**Purpose:**

Instability of the knee is a common finding in patients with congenital limb deficiency. The instability can be attributed to soft tissue abnormalities, frontal, sagittal or rotational deformity of the lower limb and bony dysplasia of the patella or of the femoral condyles. In most of the cases, these pathomorphologic changes stay asymptomatic in daily activity. However, instability can appear during deformity correction and bone-lengthening procedures, leading to flexion contracture or subluxation of the knee.

**Methods:**

A review of pediatric orthopaedic literature on different factors of knee instability, state-of-the-art treatment options in congenital limb deficiency and in cases of lengthening-related knee subluxation is presented and the authors’ preferred treatment methods are described.

**Results:**

Leg lengthening and deformity correction in patients with congenital limb deficiencies can be achieved with various techniques, such as guided growth, monolateral or circular external fixation and intramedullary lengthening nails. Radiographic assessment and clinical examination of the knee stability are obligatory to estimate the grade of instability prior to surgical procedures. Preparatory surgery, as well as preventive measures such as bracing, bridging of the knee and intensive physical therapy, can help to avoid subluxation during lengthening in unstable knees.

**Conclusions:**

Adequate surgical techniques, preventive measures and early detection of signs of subluxation can lead to good functional results in patients with congenital limb deficiency.

## Introduction

An unstable knee can be observed in various congenital deformities and appears to be one of biggest challenges in deformity correction and lengthening.

Congenital deformities which result in knee instability are longitudinal deficiencies such as congenital femoral deficiency (CFD) and fibular hemimelia (FH) or tibial hemimelia. Depending on the severity of the deficiency, the instability can be detected in early infancy or later during life, sometimes without clinical significance.

The correction of axial malalignment and leg length discrepancy is crucial in the treatment of patients with congenital longitudinal deficiencies. Severe deformities such as valgus or varus deviations, as well as rotational malalignment, can exaggerate knee instability and maltracking of the patella.

Children with severe CFD and FH may need multiple deformity correction or lengthening procedures during growth to reduce axial malalignment and leg length discrepancy. Various methods such as monolateral fixators, circular hexapod fixators and intramedullary lengthening nails have been developed. However, irrespective of the applied lengthening method, knee instability in congenital limb deficiency can unmask and can lead to knee subluxation as a severe complication.

Prevention of subluxation during lengthening in patients with congenital knee instability can be achieved with bridging of the knee with monolateral or circular fixators. Intensive physical therapy and bracing may prevent knee subluxation during lengthening using intramedullary nails.

As mild knee subluxation can easily go unrecognised on X-rays [1], clinical examination and accurate radiographic analysis during bone lengthening is necessary to detect signs of subluxation of adjacent joints as early as possible. Knee stability as well as early detection and treatment of knee subluxation has the highest priority in lengthening procedures in all patients with CFD and/or FH.

## Knee pathoanatomy

Common classifications of congenital limb deficiency are the classifications according to Paley and Standard [2] and Pappas [3] for CFD and the classification of Achterman and Kalamchi [4] for FH.

In CFD and FH, specific pathomorphologic changes of the knee have been described [5]. Potential anomalies are hypoplasia or aplasia of the anterior cruciate ligament (ACL), the posterior cruciate ligament (PCL), meniscoid changes, hypoplasia of the lateral condyle and the tibial spine and patella hypoplasia. According to the main pathology, the instability can affect the sagittal, frontal or rotational plane of the knee.

Some authors estimate that 95% of FH have an absent ACL [6]. Abnormalities of the cruciate ligaments have been verified by magnetic resonance imaging (MRI) studies [7–10] and by arthroscopy [7, 11].

Manner et al. [9] developed a classification to assess the aplasia of cruciate ligaments by tunnel view knee radiographs in patients with congenital limb deficiency. They found three types of patterns of ACL/PCL involvement in MRI and radiographs analysing the lateral and medial tibial spine and the notch width and height. The ACL was affected in all (34) knees. Hypoplasia of the ACL in combination with a normal PCL occurred in 15% and aplasia of the ACL with a normal PCL in 41%. Aplasia of the ACL and hypoplasia of the PCL was seen in 21% and total absence of both cruciate ligaments was observed in 24% [9].

Other authors made similar findings regarding the ACL and PCL changes in CFD and FH [8, 10].

Besides these pathomophologic changes within the knee, the knee stability is also influenced by axial malalignment with severe valgus deformity increasing maltracking of the patella and hyperlaxity of the overused medial collateral ligaments.

## Knee function

It remains unclear which patients with CFD or FH suffer from knee instability in daily activity or mild sports activity. Despite the pathoanatomy and signs of instability in physical examination, many authors conclude that there might be no or only insignificant instability in **daily activity** in most of the cases of CFD and FH [7, 8, 11, 12].

Others state that the **congenital cruciate aplasia** may lead to retropatellar pain and meniscus injury [12], as well as giving way during weightbearing and the development of degenerative changes.

There is not much data on functional outcome in daily activity or in sports participation of patients with congenital limb deficiency. Crawford et al. [6] presented the long-term results of 23 patients with fibular hemimelia and associated cruciate deficiency with a mean follow up of 18.6 years. They observed that patients with fibular hemimelia and cruciate deficiency can live active lives and many are able to participate in sports such as skiing, football or baseball similar to an age-matched control group. The authors state that, compared to outcome reports of traumatic ACL ruptures, patients with congenital ACL deficiency have less instability.

However, depending on the severity of the knee instability in congenital deformity, different problems can occur:

- Instability in daily activity or sports.
- Knee pain, meniscal damage or degenerative changes.
- Complications during bone lengthening or deformity correction (flexion contracture or knee subluxation).
- Recurrence of axial malalignment after deformity correction [13].

## Treatment of knee instability in congenital limb deficiency

### Arthroscopic ACL reconstruction

Reports on arthroscopic **ACL reconstruction** in congenital deficiencies of the lower limb are rare. This might be mainly because most of the patients do not complain of instability in daily activity. Furthermore, intraarticular ACL reconstruction in the absence of a femoral notch is challenging.

Gabos et al. [14] described the results of ACL reconstruction with allograft in four adolescents with congenital limb deficiency. The mean age at operation was 15.8 years. All four patients had prior limb alignment procedures and were symptomatic with instability with walking, despite of the use of an ACL brace and intensive physical therapy with muscle strengthening. The authors conclude that, with appropriate patient selection, ACL reconstruction can be an effective procedure.

Figueroa et al. [15] presented a case report of a 16-year-old patient with FH with a knee subluxation combined with a valgus and recurvatum deformity after tibial lengthening. They performed a corrective osteotomy for angular deformity correction with tibial and femoral osteotomies and simultaneous arthroscopic ACL reconstruction and observed good function at 12 months follow up.

As severe knee instability is treated with comprehensive knee reconstruction in early childhood (superknee procedure, preparatory surgery), the indication for arthroscopic ACL repair in congenital cases is rare. In the older child with symptomatic knee instability, other reasons for instability such as axial malalignment have to be corrected before considering a cruciate ligament repair.

### Preparatory surgery for lengthening procedures

Knee stability can be reconstructed in early age with complex reconstruction surgery of the knee to achieve good stability for daily activity and for future lengthening procedures. This **preparatory surgery** is often done before the age of 4 years [16].

A multitude of soft tissue procedures to reconstruct knee stability have been developed.

**Fig. 1:**
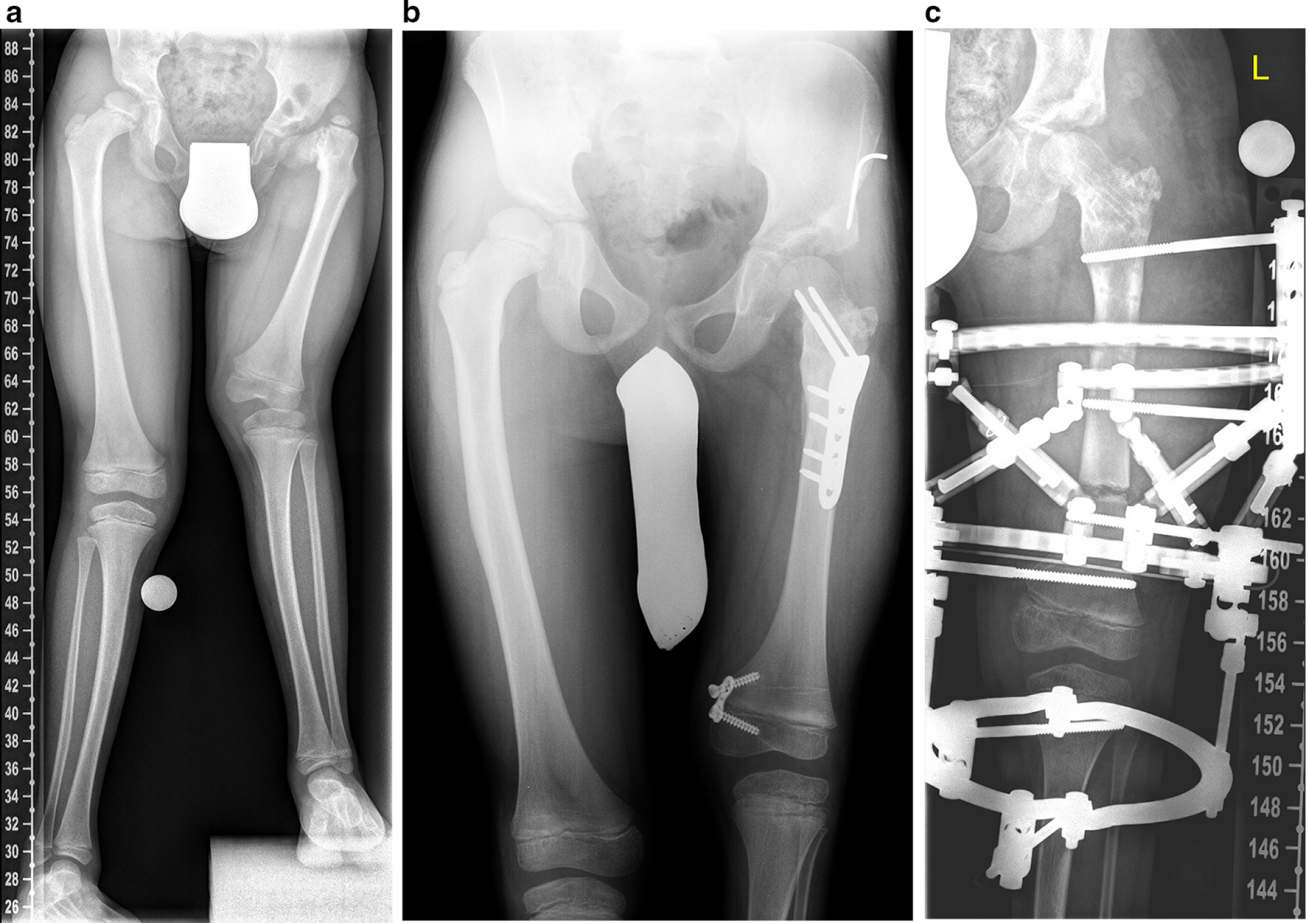
This patient with congenital femoral deficiency (CFD) and fibular hemimelia (FH) with severe genu valgum and an unstable knee and hip (**a**) underwent superhip and superknee procedures combined with guided growth as preparatory surgery for lengthening (**b**). Due to severe knee instability, a circular fixator (Taylor spatial frame, TSF) with bridging of the knee was applied for femur lengthening (**c**)

The most comprehensive procedure in congenital knee instability is the **superknee procedure** described by Paley and Standard [2]. This method comprises various soft tissue procedures and, therefore, addresses different factors of instability within the knee. The patellar stabilisation is reconstructed by lateral release of the capsule, a Grammont procedure with medialisation of the patellar tendon and a modified Langenskiöld procedure can be added if necessary. The ACL is reconstructed with a MacIntosh intra- or extraarticluar ACL (or both cruciate ligaments) repair. A soft tissue release of the iliotibial band may be performed additionally. The superknee procedure can be combined with superhip [2] and superankle procedures to treat instabilities of adjacent joints (Fig. 1a–c).

### External fixation

Various **external fixation** devices for deformity correction and bone lengthening have been developed.

Grill and Dungl [17] reported on 37 patients with congenital short femur which were treated with femoral lengthening with the Ilizarov frame (Smith and Nephew, Memphis, TN, USA) or Orthofix (Orthofix, Verona, Italy) monolateral lengthening device. Complications and knee subluxation occurred in 21%.

**Hexapod systems** for external fixators such as the Taylor spatial frame (TSF, Smith and Nephew, Memphis, TN, USA) or the Ortho-SUV frame (Ortho-SUV Ltd., St. Petersburg, Russian Federation) enable more accurate deformity correction than the Ilizarov frame [18, 19]. However, to prevent subluxation during lengthening, the knee joint still has to be bridged by the external fixator.

The safest way of lengthening is, therefore, the preventive **bridging of the knee** with a circular frame (Ilizarov, TSF). An accurately placed hinge at the centre of rotation of the knee between the femoral and tibial frame allows safe lengthening and enables controlled mobilisation of the knee joint (Fig. 1c).

The long-term results of Ilizarov or TSF lengthening in children with mild to moderate fibular hemimelia showed good results and patient satisfaction at a mean follow up of 15 years. The authors extended the frame above the knee if the knee was clinically unstable [20].

**Fig. 2:**
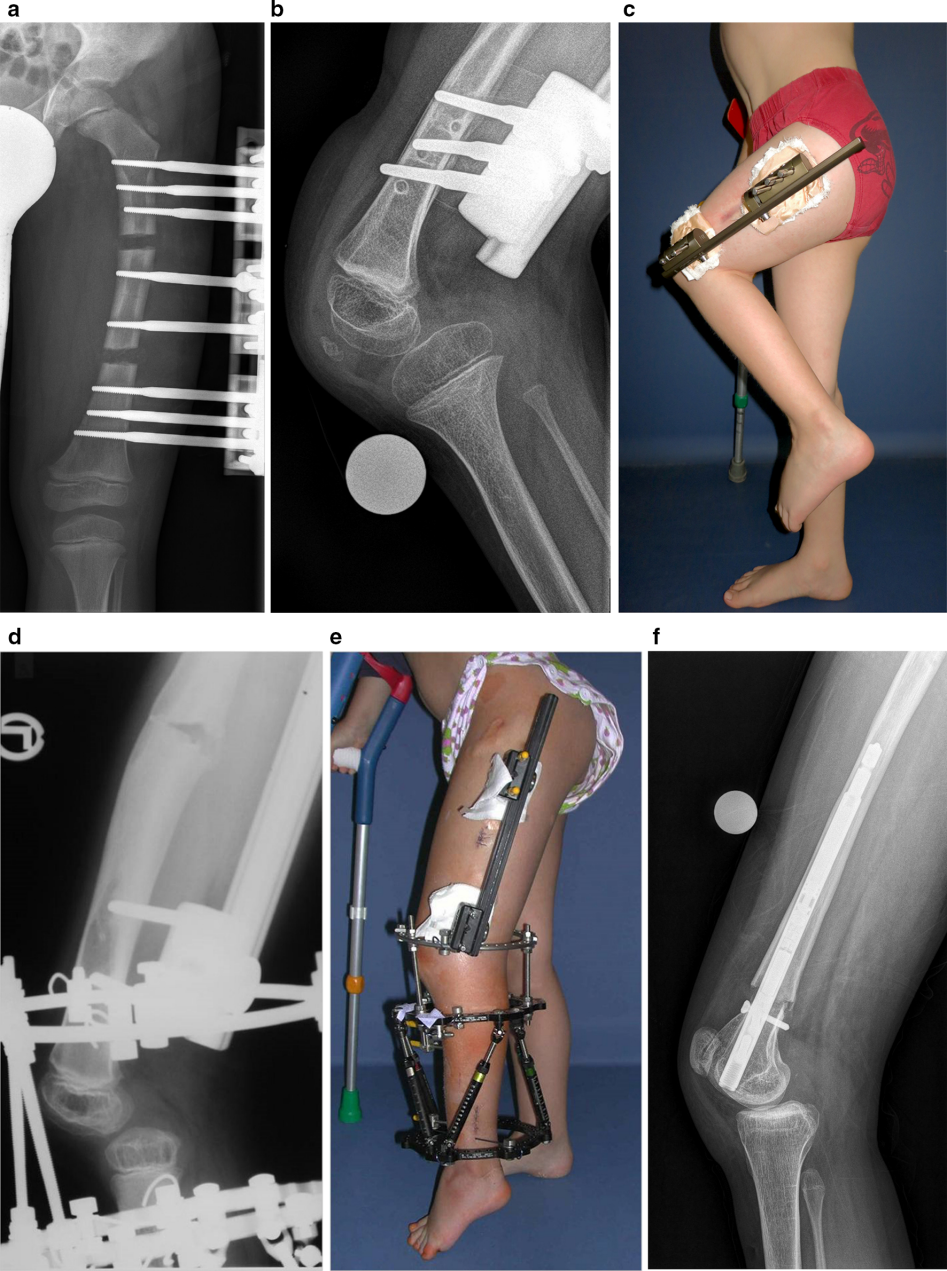
Knee subluxation can occur during bone lengthening with different devices: monolateral fixator with double osteotomy and without bridging of the knee with persistent knee subluxation (treatment performed elsewhere) (**a**–**c**); combination of monolateral and circular fixator with insufficient bridging of the knee (**d**, **e**); early knee subluxation in a case of axis correction and femoral lengthening with a retrograde PRECICE nail in a 17-year-old girl with CFD and FH (**f**)

**Monolateral fixators** for lengthening in patients with CFD and/or FH might give less stability, but with an adequate technique, the knee can be bridged as well. Again, the precise placement of the hinge at the rotational centre of the knee is essential. Without (Fig. 2a–c) or with insufficient (Fig. 2d, e) bridging of the knee, the risk of subluxation is high.

Femoral lengthening of mild and moderate CFD with clinical stable knee joints with monolateral external fixator showed good results and high function and satisfaction (PODCI score). Knee subluxation occurred in 13% and bridging of the knee and the use of an articulated external fixator was recommended [21].

Especially in the most severe knee deformity with aplasia of the cruciate ligament and ball and socket joint configuration, the frontal plane instability can lead to recurrence of axial malalignment after deformity correction [13].

### Intramedullary lengthening nails

In moderate deformities with no or mild clinical instability, **motorised intramedullary lengthening nails** can be a preferable option.

The nails show faster rehabilitation with higher knee range of motion, better callus formation and less complications compared to external fixation [22].

The development of new motorised intramedullary lengthening nails enables deformity correction and limb lengthening with higher patient satisfaction and comfort than with external fixators [23].

The use in congenital limb deficiency has been described for the FITBONE nail (Wittenstein Intens GmbH, Igersheim, Germany) [22, 24–26], the PRECICE nail (NuVasive, San Diego, CA, USA) [16, 27–29] and the Phenix nail (Phenix Medical, Paris, France) [30].

The limitations of lengthening nails should be kept in mind, as severe cruciate aplasia and joint incongruity (ball and socket joint) can lead to subluxation. Cases of subluxation during lengthening with motorised intramedullary lengthening nails have been reported [16, 28].

Shabtai et al. [16] presented the results of 21 patients with congenital leg length discrepancy treated with the PRECICE lengthening nail and a preventive brace. Three of the 21 patients had prior complex knee reconstruction as preparation for further lengthening procedures. One patient had a subluxation of the knee during lengthening.

**Fig. 3:**
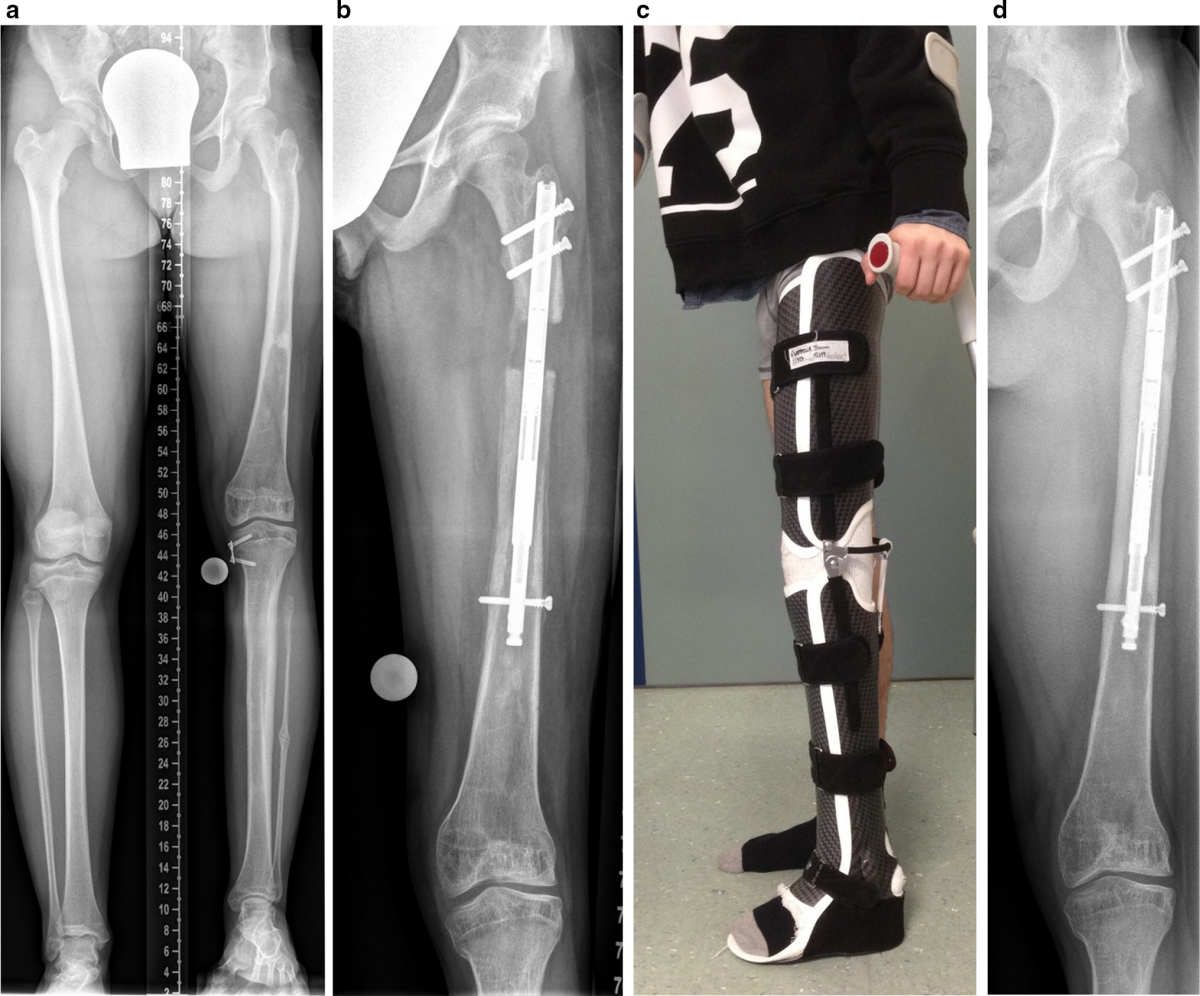
This 16-year-old female patient with CFD and FH had multiple prior surgical procedures for deformity correction and hip and knee stability. The remaining growth potential was successfully used for guided growth (**a**) before an antegrade PRECICE nail was inserted (**b**). To prevent the knee from subluxation, a custom-made knee extension brace was used and the patient attended physical therapy (**c**). Lengthening of 3 cm could be completed without knee subluxation (**d**)

We use the PRECICE nail in congenital deformity without or with minor instability in combination with an above-knee orthosis (Fig. 3c) and weekly radiographic follow up. Since 2013, a total of 57 PRECICE nails have been implanted in 54 patients. Eight of these patients had CFD and/or FH with leg length discrepancy. One patient had early subluxation of the knee despite the use of a knee extension brace (Fig. 2f).

**Guided growth** can be particularly helpful as a joint-sparing procedure to reduce the extent of other more invasive surgical techniques (Figs. 1b and 3a).

A noteworthy disadvantage of the PRECICE nail is high hardware costs. However, the nail gives various options such as retro- or antegrade femoral approach, trochanteric or piriformis femoral entry point and is available in different diameters and nail lengths. Therefore, it enables us to choose an optimal nail and surgical approach for each case.

Nevertheless, knee instability remains a huge concern in lengthening with nails. We recommend day- and night-time use of an extension brace during lengthening with an intramedullary lengthening nail. However, there are no studies on the effectiveness, optimal type (inclusion of ankle joint or pelvis) and wearing time of these braces. In the presence of significant knee instability in lower limb deficiency, lengthening with external fixation and bridging of the knee remains the gold standard of treatment.

### In case of lengthening-related subluxation

The prediction of an individual’s risk for subluxation in patients with CFD/FH remains difficult or impossible. It is necessary to detect **signs of subluxation** as early as possible to prevent the knee from sustaining severe damage.

Knee range of motion during lengthening procedures of the femur with external fixation is limited during treatment and needs several months to recover [31]. **Flexion contracture** of the knee can occur during lengthening and can be resolved by decreasing the lengthening rate or intensive physical therapy in most of the cases. However, acute or severe flexion contracture in congenital limb deficiency may indicate knee subluxation and should be followed closely.

In the case of severe flexion deformity or subluxation, it is advisable to temporarily **stop the lengthening procedure**. An existing bridging of the joint should be re-evaluated to ensure the correct position and configuration.

Motorised nails such as the PRECICE [16] and Phenix nails [30], as well as most of the external fixators, allow controlled **reverse programmes (shortening)**. Acute shortening might be advisable in severe and especially in acute subluxation to manage that complication.

**Conservative treatment** with physical therapy [21, 28] and the use of a knee brace [28, 32] was reported to reduce or resolve knee flexion contracture and knee subluxation in cases of lengthening-related subluxation.

Furthermore, various **surgical procedures** such as simultaneous corrective osteotomy with arthroscopic ACL repair [15], PCL reconstruction [21], soft tissue release and ligament reconstruction [16] to treat lengthening-related subluxation or knee flexion contracture have been described. In severe cases, external fixation may be necessary to realign the tibiofemoral alignment [1, 17]. Most of these reports are case reports with various treatment regimens and with limited clinical follow up, which precludes the development of further treatment guidelines.

**Consequences of subluxation** and residual knee flexion deformity can be severe with functional leg length discrepancy (despite the prior lengthening procedure), anterior knee pain and inability to weight bear due to knee instability (Fig. 2c).

## Conclusion

Knee joints of patients with congenital limb deficiencies show individually highly variable grades of instability. The knee function in daily activity seems to be nearly normal, even with knee abnormalities such as cruciate ligament aplasia and dysplasia of the joint surfaces. The instability might be relevant in sports activity, but further studies on this topic are needed. However, special attention is necessary prior to lengthening and deformity correction. Preoperative radiographic assessment is essential and clinical assessment of instability is obligatory. The development of new lengthening devices enabled us to correct congenital limb deficiencies with higher patient satisfaction and comfort. Irrespective of the applied method, high functional outcome can only be achieved by obeying the general rules of leg lengthening, with the highest aim of preventing the joint sustaining any damage during the lengthening procedure. In cases of severe flexion contracture or knee subluxation during lengthening, we recommend to stop lengthening or transiently shorten the bone to protect the knee joint from further damage. Intensive physical therapy and the use of a knee extension brace can help to prevent and treat subluxation. Adequate surgical techniques, preventive measures and early detection of signs of subluxation can lead to good functional results in patients with congenital limb deficiency.
